# Expanding the clinical phenotype of individuals with a 3-bp in-frame deletion of the *NF1* gene (c.2970_2972del): an update of genotype–phenotype correlation

**DOI:** 10.1038/s41436-018-0269-0

**Published:** 2018-09-07

**Authors:** Magdalena Koczkowska, Tom Callens, Alicia Gomes, Angela Sharp, Yunjia Chen, Alesha D. Hicks, Arthur S. Aylsworth, Amedeo A. Azizi, Donald G. Basel, Gary Bellus, Lynne M. Bird, Maria A. Blazo, Leah W. Burke, Ashley Cannon, Felicity Collins, Colette DeFilippo, Ellen Denayer, Maria C. Digilio, Shelley K. Dills, Laura Dosa, Robert S. Greenwood, Cristin Griffis, Punita Gupta, Rachel K. Hachen, Concepción Hernández-Chico, Sandra Janssens, Kristi J. Jones, Justin T. Jordan, Peter Kannu, Bruce R. Korf, Andrea M. Lewis, Robert H. Listernick, Fortunato Lonardo, Maurice J. Mahoney, Mayra Martinez Ojeda, Marie T. McDonald, Carey McDougall, Nancy Mendelsohn, David T. Miller, Mari Mori, Rianne Oostenbrink, Sebastién Perreault, Mary Ella Pierpont, Carmelo Piscopo, Dinel A. Pond, Linda M. Randolph, Katherine A. Rauen, Surya Rednam, S. Lane Rutledge, Veronica Saletti, G. Bradley Schaefer, Elizabeth K. Schorry, Daryl A. Scott, Andrea Shugar, Elizabeth Siqveland, Lois J. Starr, Ashraf Syed, Pamela L. Trapane, Nicole J. Ullrich, Emily G. Wakefield, Laurence E. Walsh, Michael F. Wangler, Elaine Zackai, Kathleen B. M. Claes, Katharina Wimmer, Rick van Minkelen, Alessandro De Luca, Yolanda Martin, Eric Legius, Ludwine M. Messiaen

**Affiliations:** 1 Department of Genetics, University of Alabama at Birmingham, Birmingham, Alabama USA; 2 Departments of Pediatrics and Genetics, University of North Carolina at Chapel Hill, Chapel Hill, North Carolina USA; 3 Division of Neonatology, Pediatric Intensive Care and Neuropediatrics, Department of Pediatrics and Adolescent Medicine, Medical University of Vienna, Vienna, Austria; 4 Children’s Hospital of Wisconsin, Milwaukee, Wisconsin USA; 5 Department of Clinical Genetics and Metabolism, Children’s Hospital, University of Colorado School of Medicine, Aurora, Colorado USA; 6 Department of Pediatrics, University of California San Diego; Division of Genetics/Dysmorphology, Rady Children’s Hospital, San Diego, California USA; 7 Baylor Scott and White Hospital, Temple, Texas USA; 8 Clinical Genetics Program, University of Vermont Medical Center, Burlington, Vermont USA; 9 Department of Clinical Genetics, The Children’s Hospital at Westmead, Westmead, New South Wales Australia; 10 Department of Pediatrics, Division of Genomic Medicine, UC Davis MIND Institute, Sacramento, California USA; 11 Department of Human Genetics, KU Leuven–University of Leuven, Leuven, Belgium; 12 Medical Genetics Unit, Bambino Gesù Children’s, IRCCS, Rome, Italy; 13 Carolinas Medical Center, Charlotte, North Carolina USA; 14 SOC Genetica Medica, AOU Meyer, Florence, Italy; 15 Department of Neurology, Division of Child Neurology, University of North Carolina School of Medicine, Chapel Hill, North Carolina USA; 16 Neurofibromatosis Diagnostic & Treatment Program, St. Joseph’s Children’s Hospital, Paterson, New Jersey USA; 17 Neurofibromatosis Program, Children’s Hospital of Philadelphia, Philadelphia, Pennsylvania USA; 18 Department of Genetics, Hospital Universitario Ramón y Cajal, Institute of Health Research (IRYCIS), Madrid, Spain; 19 Center for Biomedical Research-Network of Rare Diseases (CIBERER), Madrid, Spain; 20 Center for Medical Genetics, Ghent University Hospital, Ghent, Belgium; 21 Department of Neurology and Cancer Center, Massachusetts General Hospital, Boston, Massachusetts USA; 22 Division of Clinical and Metabolic Genetics, The Hospital for Sick Children, Toronto, Ontario Canada; 23 Department of Molecular and Human Genetics, Baylor College of Medicine, Houston, Texas USA; 24 Department of Pediatrics, Northwestern University Feinberg School of Medicine, Chicago, Illinois USA; 25 Medical Genetics Unit, G. Rummo Hospital, Benevento, Italy; 26 Department of Genetics, Yale University, New Haven, Connecticut USA; 27 Division of Genetics and Genomics, Boston Children’s Hospital, Boston, Massachusetts USA; 28 Department of Pediatrics, Division of Medical Genetics, Duke University School of Medicine, Durham, North Carolina USA; 29 Division of Human Genetics, Children’s Hospital of Philadelphia, Philadelphia, Pennsylvania USA; 30 Genomics Medicine Program, Children’s Hospital Minnesota, Minneapolis, Minnesota USA; 31 Department of Pediatrics, Warren Alpert Medical School, Brown University, Providence, Rhode Island USA; 32 Department of General Pediatrics, Erasmus MC-Sophia, Rotterdam, The Netherlands; 33 CHU Sainte-Justine, Mother and Child University Hospital Center, Montréal, Québec Canada; 34 Department of Pediatrics and Ophthalmology, University of Minnesota, Minneapolis, Minnesota USA; 35 U.O.S.C. Medical Genetics, A.O.R.N. “A. Cardarelli”, Naples, Italy; 36 Division of Medical Genetics, Children’s Hospital Los Angeles, Keck School of Medicine, University of Southern California, Los Angeles, California USA; 37 Department of Pediatrics, Section of Hematology-Oncology, Baylor College of Medicine, Houston, Texas USA; 38 Developmental Neurology Unit, IRCCS Foundation, Carlo Besta Neurological Institute, Milan, Italy; 39 Division of Medical Genetics, University of Arkansas for Medical Sciences, Arkansas Children’s Hospital, Little Rock, Arkansas USA; 40 Division of Human Genetics, Cincinnati Children’s Hospital Medical Center, Cincinnati, Ohio USA; 41 Genetic Medicine, Munroe-Meyer Institute, University of Nebraska Medical Center, Omaha, Nebraska USA; 42 DCH Regional Medical Center and Northport Medical Center, Northport, Alabama USA; 43 Stead Family Department of Pediatrics, University of Iowa Hospitals & Clinics, Iowa City, Iowa USA; 44 Department of Neurology, Boston Children’s Hospital, Boston, Massachusetts USA; 45 Department of Neurology, Indiana University School of Medicine, Indianapolis, Indiana USA; 46 Division of Human Genetics, Medical University of Innsbruck, Innsbruck, Austria; 47 Department of Clinical Genetics, Erasmus Medical Center, Rotterdam, The Netherlands; 48 IRCCS Casa Sollievo della Sofferenza, Molecular Genetics Unit, San Giovanni Rotondo, Foggia, Italy

**Keywords:** NF1, p.Met992del, genotype–phenotype correlation, neurofibroma, learning difficulties

## Abstract

**Purpose:**

Neurofibromatosis type 1 (NF1) is characterized by a highly variable clinical presentation, but almost all NF1-affected adults present with cutaneous and/or subcutaneous neurofibromas. Exceptions are individuals heterozygous for the *NF1* in-frame deletion, c.2970_2972del (p.Met992del), associated with a mild phenotype without any externally visible tumors.

**Methods:**

A total of 135 individuals from 103 unrelated families, all carrying the constitutional *NF1* p.Met992del pathogenic variant and clinically assessed using the same standardized phenotypic checklist form, were included in this study.

**Results:**

None of the individuals had externally visible plexiform or histopathologically confirmed cutaneous or subcutaneous neurofibromas. We did not identify any complications, such as symptomatic optic pathway gliomas (OPGs) or symptomatic spinal neurofibromas; however, 4.8% of individuals had nonoptic brain tumors, mostly low-grade and asymptomatic, and 38.8% had cognitive impairment/learning disabilities. In an individual with the *NF1* constitutional c.2970_2972del and three astrocytomas, we provided proof that all were NF1-associated tumors given loss of heterozygosity at three intragenic *NF1* microsatellite markers and c.2970_2972del.

**Conclusion:**

We demonstrate that individuals with the *NF1* p.Met992del pathogenic variant have a mild NF1 phenotype lacking clinically suspected plexiform, cutaneous, or subcutaneous neurofibromas. However, learning difficulties are clearly part of the phenotypic presentation in these individuals and will require specialized care.

## Introduction

Neurofibromatosis type 1 (NF1, MIM 162200) is an autosomal dominant disorder affecting 1 in every 2000 to 3000 births with age-dependent penetrance and highly variable inter- and intrafamilial expressivity.^1^ The main clinical signs of NF1 include café-au-lait macules (CALMs), skinfold freckling, Lisch nodules, neurofibromas, optic pathway gliomas (OPGs), and/or specific skeletal abnormalities, such as sphenoid wing or tibial dysplasia. According to the diagnostic criteria established by the National Institutes of Health (NIH), the presence of at least two of the aforementioned features (or a single clinical symptom with a first-degree relative meeting NIH criteria) is sufficient for a clinical diagnosis of NF1^2^. The NIH diagnostic criteria are highly specific and sensitive in NF1-affected adults as nearly all have cutaneous and/or subcutaneous neurofibromas.^3^ Examples of individuals consistently presenting only with typical pigmentary manifestations (multiple CALMs with/without freckling) without externally visible plexiform, cutaneous, or subcutaneous neurofibromas are these heterozygous for the *NF1* deletion p.Met992del and missense pathogenic variants at residue p.Arg1809^4–6^. Because NF1 is a progressive disorder with phenotypic manifestations increasing with age, the development of serious complications still needs consideration in individuals with p.Met992del until more data, especially in adults, have become available. The association of p.Met992del with a mild phenotype was originally established based on the analysis of 21 unrelated probands and 26 affected relatives, with only 19 individuals ≥19 years old.^4^ Larger data sets, preferably of adults, are required to confirm the mild clinical course in these individuals and identify rare complications.

We describe in this study detailed phenotypic characterization of an additional 135 individuals from 103 unrelated families carrying the *NF1* p.Met992del pathogenic variant. Besides confirming the absence of superficial neurofibromas, we demonstrate that some complications, such as nonoptic brain tumors and cognitive impairment and/or learning disabilities, are present in a significant number of these individuals (4.8% and 38.8%, respectively). We also provide proof of the existence of low-grade pilocytic astrocytomas with the *NF1* p.Met992del pathogenic variant and loss of heterozygosity (LOH) at p.Met992del and three intragenic *NF1* microsatellite markers in three lesions from a single NF1-affected individual, establishing these as NF1-associated tumors.

## Materials and methods

A total of 135 individuals from 103 unrelated families were referred to the Medical Genomics Laboratory at University of Alabama at Birmingham (UAB; 74 probands and 27 affected relatives) and collaborating centers (EUR; 29 probands and 5 affected relatives) for *NF1* molecular testing (see details in Table S1). All were confirmed as having the same *NF1* 3-bp in-frame deletion c.2970_2972del, leading to loss of a methionine at codon 992 (p.Met992del), except for a 14-month-old girl heterozygous for the *NF1* c.2974_2976del, also resulting in p.Met992del (UAB-R7536). Comprehensive *NF1* molecular analysis with an RNA-based approach was performed in the Medical Genomics Laboratory as previously described,^6,7^ with LRG_214 and NM_000267.3 used as the reference sequences.

Clinical data were collected as previously reported^6,7^ at the time of genetic testing with data reverified by referring physicians for accuracy and/or updating, when available. As magnetic resonance imaging (MRI) is not routinely performed in individuals without clinical signs/suspicion for spinal neurofibromas and/or OPGs, presence of internal neurofibromas and asymptomatic OPGs could not be excluded in all cases. If ≥2 Noonan-like features (i.e., short stature, facial dysmorphism [low-set ears, hypertelorism, downslanted palpebral fissures, midface hypoplasia, ptosis, and/or webbed neck] or cardiac abnormality [pulmonic stenosis]) were present, an individual was classified as having Noonan-like phenotype. Short stature and macrocephaly were defined as previously described.^6,7^

We compared the phenotypes of individuals with the *NF1* p.Met992del pathogenic variant with the cohorts of individuals heterozygous for the *NF1* missense pathogenic variants affecting codons 1809 and 844–848 ^5–10^, and previously described large-scale cohorts with “classic” NF1^11–24^. Two-tailed Fisher’s exact test with *P* values adjusted for multiple comparisons using Benjamini–Hochberg (B–H) procedure with false discovery rates (FDR) at 0.05 and 0.01 was applied (GraphPad software; Table S2).

This study was approved by the institutional review boards of all participating institutions offering clinical genetic testing.

## Results

### Clinical description of the studied group

Among 103 unrelated probands, 38/103 (36.9%) were familial and 43/103 (41.8%) were sporadic cases, with 6/43 individuals proven to have a de novo variant, but no formal paternity/maternity testing was performed. Two individuals with proven de novo p.Met992del (UAB-R6151 and UAB-R2586) had one parent reported to have 1–5 CALMs with/without freckling; thus gonosomal mosaicism or the presence of a different independent *NF1* pathogenic variant cannot be excluded in these families (Table S1). For 22/103 (21.4%) cases family history was unknown, including three adopted probands.

Ninety-seven of 131 individuals (74.1%) fulfilled the NIH diagnostic criteria, but only 76/131 (58%) if family history was excluded as a criterion (Table 1). Of the 55 cases not fulfilling the NIH diagnostic criteria after excluding the family history, 31 had complete clinical information including the ophthalmological results for the presence/absence of Lisch nodules and symptomatic OPGs (Table S1 and Table S3), including 11/31 (35.5%) children ≤8 years old, 10/31 (32.3%) individuals between 9 and 18 years old, and 10/31 (32.3%) adults over 19 years old. Five of 31 individuals had <6 CALMs as the only clinical feature (Table S3).

**Table 1:** Demographic and clinical characterization of individuals with the *NF1* p.Met992del pathogenic variant

NF1 feature	*N* (%)			
	≤8 years	9–18 years	≥19 years	All ages
Mutation-positive individuals [proband:relative]	45 [41:4]	50 [43:7]	40 [19:21]	135 [103:32]
Male:female	15:30	27:23	18:22	60:75
Fulfilling the NIH criteria if the family history is taken into account	31/43 (72.1)	43/49 (87.8)	23/39 (59)	97/131 (74.1)
Fulfilling the NIH criteria if solely taking the physical signs into account	20/43 (46.5)	36/49 (73.5)	20/39 (51.3)	76/131 (58)
>5 CALMs	41/45 (91.1)	48/50 (96)	30/40 (75)^h^	119/135 (88.2)
Skinfold freckling	20/39 (51.3)	35/48 (72.9)	18/37 (48.7)	73/124 (58.9)
Lisch nodules	3/34 (8.8)	6/43 (14)	4/24 (16.7)	13/101 (12.9)
Skeletal abnormalities^a^	5/39 (12.8)	9/48 (18.8)	7/38 (18.4)	21/125 (16.8)
Major external plexiform neurofibromas	0/44 (0)	0/46 (0)	0/38 (0)	0/128 (0)
Cutaneous neurofibromas^b^	0/43 (0)	0/47 (0)	0–1/38 (0–2.6)^i^	0–1/128 (0–0.8)
Subcutaneous neurofibromas^b^	0/42 (0)	0/46 (0)	0–3/36 (0–8.3)^i^	0–3/124 (0–2.4)
Symptomatic spinal neurofibromas	0/39 (0)	0/45 (0)	0/34 (0)	0/118 (0)
Symptomatic OPGs^c^	0/43 (0)	0/47 (0)	0/33 (0)	0/123 (0)
Asymptomatic OPGs^d^	0/11 (0)	1/19 (5.3)	0/11 (0)	1/41 (2.4)
Other neoplasms^e^	0/42 (0)	5/47 (10.6)	8/37 (21.6)	13/126 (10.3)
Cognitive impairment and/or learning disabilities	14/42 (33.3)	24/49 (49)	12/38 (31.6)	50/129 (38.8)
Noonan-like features^f^	3/42 (7.1)	6/43 (14)	6/34 (17.7)	15/119 (12.6)
Short stature^g^	2/24 (8.3)	6/32 (18.8)	3/15 (20)	11/71 (15.5)
Macrocephaly	12/32 (37.5)	8/40 (20)	6/15 (40)	26/87 (29.9)
Pulmonic stenosis	3/38 (7.9)	1/44 (2.3)^j^	0/31 (0)	4/113 (3.5)

*CALMs*, café-au-lait macules; *NF1*, neurofibromatosis type 1; *NIH*, National Institutes of Health; *OPG*, optic pathway glioma.

^a^All bone abnormalities included, that is, scoliosis (*n* = 11), pectus abnormality (*n* = 9), mild winging of the scapula (*n* = 1), rib abnormality (*n* = 1), dysplastic vertebrae (*n* = 1), kyphosis (*n* = 1), and bilateral club feet (*n* = 1).

^b^At least two cutaneous/subcutaneous neurofibromas were required to be considered as “positive for the criterion of neurofibromas.”

^c^The absence of symptomatic OPGs was determined by ophthalmological examination and/or by magnetic resonance image (MRI).

^d^Including only individuals without signs of symptomatic OPGs who underwent MRI examination.

^e^All “other” neoplasms, not including OPGs and neurofibromas, included, that is, astrocytomas (*n* = 3), oligodendroglioma (*n* = 1), lipomas (*n* = 5), angiolipoma (*n* = 1), hypothalamic glioma (*n* = 1), brain tumor with hamartomatous aspect by MRI of the encephalon (*n* = 1), neuroblastoma (*n* = 1), and craniopharyngioma (*n* = 1).

^f^An individual was classified as having Noonan-like phenotype when at least two of the following features were present: short stature, low-set ears, hypertelorism, downslanted palpebral fissures, midface hypoplasia, ptosis, webbed neck, and/or pulmonic stenosis.

^g^As no specific growth curves are available for the Hispanic and Asian populations, Hispanic and Asian individuals were excluded as having short or normal stature.

^h^A possible explanation for a decreasing prevalence of CALMs in individuals ≥19 years is the fact that CALMs become fainter with age and some may even disappear entirely.

^i^Four individuals with few (2–6) cutaneous or subcutaneous “neurofibromas”; none were biopsied and therefore none have been histologically confirmed.

^j^The presence of pulmonic stenosis was reported in the individual’s newborn period.

The presence of >5 CALMs and freckling was observed in 119/135 (88.2%) and 73/124 (58.9%) cases, respectively. Fifty-two of 85 individuals ≥9 years old had both pigmentary manifestations, while Lisch nodules were observed in 12.9% (13/101) of the studied cohort, including 14.9% (10/67) ≥9 years old. No symptomatic OPGs were found herein (0/123); however, of 41 asymptomatic individuals receiving brain MRI screening, a single bilateral OPG located in chiasm and optic nerves was reported (EUR-R1).

No *histopathologically* confirmed externally visible plexiform (0/128, including 0/84 individuals ≥9 years old), cutaneous (0/128, including 0/38 individuals ≥19 years), or subcutaneous neurofibromas (0/124, including 0/36 individuals ≥19 years) were found. In 14 cases ≥14 years a single or a few cutaneous or subcutaneous lesions *suspected* to be neurofibromas were observed (Table S1 and Table S4). Importantly, for five individuals the diagnosis was “lipoma” instead after detailed clinical and/or histopathological analysis. Symptomatic spinal neurofibromas were absent in all 118 individuals and the presence of asymptomatic spinal tumors was excluded by MRI in 13 cases (with 12/13 individuals ≥9 years old).

Twenty-one of 125 individuals (16.8%) had skeletal abnormalities (Table 1 and Table S1). Besides scoliosis (11/125 all ages, but 5/38 ≥19 years) and pectus anomalies (9/125), other skeletal abnormalities included rib abnormality, bilateral club feet, dysplastic vertebrae, mild winging of the scapula and kyphosis (each observed in a single individual).

Nonmalignant tumors, not including OPGs and neurofibromas, were identified in 12/126 (9.5%) individuals and included astrocytomas (*n* = 3, one tumor was described as having features of astrocytoma and dysembryoplastic neuroepithelial tumor [DNET], see details in Table S1), oligodendroglioma (*n* = 1), lipomas (*n* = 5), angiolipoma (*n* = 1), hypothalamic glioma (*n* = 1), brain tumor with hamartomatous aspect by MRI of the encephalon (*n* = 1), and craniopharyngioma (*n* = 1) (Table 1). In addition, one individual had a history of a neuroblastoma, but detailed follow-up was not available (Table S1 and Table S5). No breast cancer was observed in the studied cohort (0/20 women ≥19 years). Of particular interest was a 42-year-old male with no clinical signs of NF1 (UAB-R5571-F) and no history of prior irradiation, but with three surgically removed juvenile pilocytic astrocytomas, one located in the brainstem and two in the top left side of the brain (Table S1). He was molecularly diagnosed as being heterozygous for *NF1* p.Met992del after diagnosis was made in his son who had >5 CALMs, left inguinal freckling, macrocephaly, and abnormal development (Fig. S1). Morphologically, an astrocytic neoplasm composed of two growth patterns was observed, i.e., areas with a fibrillary arrangement and Rosenthal fibers and areas with a pattern similar to that seen in oligodendroglioma; there was no significant pleomorphism, increased mitotic activity, or necrosis. Molecular analysis revealed the *NF1* pathogenic variant c.2970_2972del with LOH at c.2970_2972del and all three intragenic *NF1* microsatellite markers tested in all three astrocytomas, confirming these as NF1-associated tumors.

Fifty of 129 case subjects (38.8%) had an abnormal development with at least one of the following forms of cognitive impairment or learning disabilities: learning difficulties (*n* = 38), developmental delay (*n* = 10), speech delay (*n* = 15), motor delay (*n* = 7), autism spectrum (*n* = 1), or psychiatric problems (*n* = 2). Of these, three individuals (UAB-R1873, UAB-R1873-M, and UAB-R6975) carried known additional genomic imbalances: 1q21.1 deletion (MIM 612474), 12q23.2 duplication, and 15q13.3 duplication (Table S1 and Fig. S2). In addition, array comparative genomic hybridization (aCGH) analysis revealed the presence of a 12.6-kb-sized deletion on 16p13 and 226.9 kb-sized duplication on 14q32.13 of unclear clinical significance in a single individual (UAB-R8603) (Table S1). Noonan-like features were found in 15/119 (12.6%) individuals, including the presence of pulmonic stenosis in four children ≤8 years old. In all 10 individuals with Noonan-like phenotype referred to UAB no pathogenic or likely pathogenic variants in 16 Noonan-related disorder genes (see details in Table S1) were identified. Other cardiovascular abnormalities included hypertension, double aortic arch with vascular ring, ventricular tachycardia, sinus arrhythmia and atypia of repolarization, and atrial septal defect (each observed in a single individual). Short stature and macrocephaly were found in 11/71 (15.5%) and 26/87 (29.9%) of case subjects, respectively.

### Comparison of clinical features in individuals heterozygous for *NF1* p.Met992del with cohorts of individuals with *NF1* missense pathogenic variants affecting codons 1809 and 844–848 and with “classic” NF1 phenotype

Individuals with p.Met992del had Lisch nodules significantly less often and no externally visible plexiform neurofibromas, cutaneous and subcutaneous neurofibromas, compared with the cohort of individuals with the *NF1* missense pathogenic variants at residues 844–848 and previously reported “classic” NF1 population (all *P* < 0.0001, statistically significant after B–H correction at FDR 0.01; Table 2). Importantly, no individuals described in this and previous studies had visible plexiform neurofibromas (0/127 ≥9 years) or *histopathologically* confirmed cutaneous (0/59 ≥19 years) or subcutaneous neurofibromas (0/37 ≥19 years) (Table S6).^4,25,26^ Similar to the *NF1* p.Arg1809 cohort, no symptomatic OPGs were observed in the studied group. In the current study, none of the individuals showed symptomatic spinal neurofibromas (Table 1), although in the original report a single symptomatic spinal tumor was found;^4^ nevertheless, even combined that remains significantly less prevalent than in the cohort of individuals with the *NF1* missense pathogenic variants at residues 844–848 (*P* < 0.0001, statistically significant after B–H correction at FDR 0.01).

**Table 2:** Comparison of clinical features of the cohort of individuals heterozygous for the *NF1* p.Met992del pathogenic variant with the cohorts of individuals with the *NF1* missense pathogenic variants affecting codons 1809 and 844–848 as well as with large-scale previously reported cohorts of individuals with “classic” NF1

NF1 feature	*N* (%)				*P* value (2-tailed Fisher’s exact test)		
	p.Met992del^a^	p.Arg1809^b^	aa 844–848^c^	Previously reported NF1 cohorts^d^	p.Met992del vs. p.Arg1809	p.Met992del vs. aa 844–848	p.Met992del vs. “classic” NF1
>5 CALMs	165/182 (90.7)	157/169 (92.9)	130/157 (82.8)	1537/1728 (89)^16^		0.0357 ↗	
Skinfold freckling	105/171 (61.4)	95/161 (59)	104/144 (72.2)	1403/1667 (84.2)^16^			<0.0001^p^ ↘
Lisch nodules	16/139 (11.5)	12/120 (10)	42/98 (42.9)	729/1237 (58.9)^16^		<0.0001^p^ ↘	<0.0001^p^ ↘
Major external plexiform neurofibromas^e^	0/125 (0)	0/105 (0)	36/92 (39.1)	120/648 (18.5)^11,18^		<0.0001^p^ ↘	<0.0001^p^ ↘
Cutaneous neurofibromas^f^	0–1/57 (0–1.8)^g^	0/57 (0)	47/69 (68.1)	656/723 (90.7)^12,13,18,22,23^		<0.0001^p^ ↘	<0.0001^p^ ↘
Subcutaneous neurofibromas^f^	0–3/36 (0–8.3)^g^	0–5/57 (0–8.8)^g^	33/65 (50.8)	297/515 (57.7)^18,22,23^		<0.0001^p^ ↘	<0.0001^p^ ↘
Symptomatic spinal neurofibromas	1/165 (0.6)	0/76 (0)	13/127 (10.2)	36/2058 (1.8)^11,18,19^		0.0001^p^ ↘	
Symptomatic OPGs^h^	0/170 (0)	0/139 (0)	12/136 (8.8)	64/1650 (3.9)^16^		<0.0001^p^ ↘	0.0033^o^ ↘
Asymptomatic OPGs^i^	1/41 (2.4)	0/38 (0)	18/63 (28.6)	70/519 (13.5)^14,21,24^		0.0005^p^ ↘	0.0473 ↘
Brain tumors	6/126 (4.8)^j^	1/129 (0.8)^k^	4/139 (2.9)^l^				
Skeletal abnormalities	30/172 (17.4)	21/126 (16.7)	48/144 (33.3)	144/948 (15.2)^11,17,18,23^		0.0016^o^ ↘	
Scoliosis^f^	7/57 (12.3)	6/48 (12.5)	20/64 (31.3)	51/236 (21.6)^12,13,23^		0.0159 ↘	
Cognitive impairment and/or learning disabilities	58/176 (33)	80/159 (50.3)	56/138 (40.6)	190/424 (44.8)^11,18^	0.0018^o^ ↘		0.0082^o^ ↘
Noonan-like features^m^	19/166 (11.5)	46/148 (31.1)	10/134 (7.5)	57/1683 (3.4)^16^	<0.0001^p^ ↘		<0.0001^p^ ↗
Short stature^n^	16/118 (13.6)	32/111 (28.8)	15/91 (16.5)	109/684 (15.9)^11,22^	0.0056^o^ ↘		
Macrocephaly	30/132 (22.7)	31/107 (29)	36/98 (36.7)	239/704 (33.9)^11,22^		0.0267 ↘	0.0111 ↘
Pulmonic stenosis	8/160 (5)	14/132 (10.6)	2/113 (1.8)	25/2322 (1.1)^20^			0.0009^p^ ↗

*CALMs*, café-au-lait macules; *NF1*, neurofibromatosis type 1; *OPG*, optic pathway glioma.

^a^Based on data from this study and Upadhyaya et al.^4^

^b^Based on data from Pinna et al.,^5^ Rojnueangnit et al.,^6^ Ekvall et al.,^8^ Nyström et al.,^9^ and Santoro et al.^10^

^c^Based on data from Koczkowska et al.^7^

^d^Based on data from Huson et al.,^11–13^ Listernick et al.,^14^ Van Es et al.,^15^ Friedman and Birch,^16^ Cnossen et al.,^17^ McGaughran et al.,^18^ Thakkar et al.,^19^ Lin et al.,^20^ Blazo et al.,^21^ Khosrotehrani et al.,^22^ Plotkin et al.,^23^ and/or Blanchard et al.^24^

^e^In individuals ≥9 years old.

^f^In individuals ≥19 years old.

^g^Individuals with few (2–6) cutaneous or subcutaneous “neurofibromas”; none were biopsied and therefore none have been histologically confirmed.

^h^The absence of symptomatic OPGs was determined by ophthalmological examination and/or by magnetic resonance imaging (MRI).

^i^Including only individuals without signs of symptomatic OPGs who underwent MRI examination.

^j^Only brain tumors, not including OPGs and neurofibromas, included, that is, astrocytoma (*n* = 1), oligodendroglioma (*n* = 1), hypothalamic glioma (*n* = 1), craniopharyngioma (*n* = 1), brain tumor with hamartomatous aspect by MRI of the encephalon (*n* = 1), and astrocytoma/dysembryoplastic neuroepithelial tumor (DNET) (*n* = 1).

^k^Only brain tumors, not including OPGs and neurofibromas, included, that is, astrocytoma (*n* = 1).

^l^Only brain tumors, not including OPGs and neurofibromas, included, that is, hypothalamic glioma (*n* = 1) and cerebral tumors (*n* = 3).

^m^An individual was classified as having Noonan-like phenotype when at least two of the following features were present: short stature, low-set ears, hypertelorism, midface hypoplasia, webbed neck, and/or pulmonic stenosis.

^n^As no specific growth curves are available for the Hispanic and Asian populations, Hispanic and Asian individuals were excluded as having short or normal stature.

**Statistically significant** ***P*** **values with false discovery rates (FDR) of 0.05 (indicated by** ^**o**^**) and 0.01 (indicated by** ^**p**^**) after correction for multiple testing using Benjamini–Hochberg procedure (see details in Table** **S2****). After applying the Benjamini–Hochberg correction,** ***P*** **≤ 0.0082 and** ***P*** **≤ 0.0009 remained statistically significant at FDR of 0.05 and 0.01, respectively. The black arrows indicate the statistically significant differences of the NF1 clinical features prevalence between the p.Met992del group and the cohort(s) used for the comparison, with the up and down arrows representing an increase and a decrease of the prevalence in the p.Met992del group, respectively.**

The mild phenotype without any externally visible neurofibromas observed in the current study is therefore similar to that of individuals with the *NF1* p.Arg1809 missense pathogenic variants, except for the presence of brain tumors being more prevalent in the p.Met992del-positive cohort (4.8% versus 0.8%), but this difference was not statistically significant (*P* = 0.0640).

The prevalence of skeletal abnormalities was similar as in the *NF1* p.Arg1809 cohort and “classic” NF1 population (17.4% versus 16.7% versus 15.2%, respectively), but lower than in the cohort of individuals with the *NF1* missense pathogenic variants at residues 844–848 (*P* = 0.0016, statistically significant after B–H correction at FDR 0.05).

Noonan-like features and pulmonic stenosis were much more prevalent in the studied group compared with the general NF1 population (*P* < 0.0001 and *P* = 0.0009, respectively, both statistically significant after B–H correction at FDR 0.01). However, compared with the p.Arg1809 cohort, individuals with p.Met992del had Noonan-like phenotype and short stature statistically less often (*P* < 0.0001 and *P* = 0.0056, statistically significant after B–H correction at FDR 0.01 and 0.05, respectively; Table 2). In addition, macrocephaly was observed less frequently than in the cohorts of individuals with the *NF1* missense pathogenic variants at residues 844–848 and “classic” NF1 clinical presentation (*P* = 0.0267 and *P* = 0.0111, respectively, not statistically significant after B–H correction at FDR 0.05; Table 2). Finally, individuals with p.Met992del had a significantly lower prevalence of cognitive impairment and/or learning disabilities compared with the cohorts of individuals with the *NF1* missense pathogenic variants at codon 1809 and the general NF1 population (*P* = 0.0018 and *P* = 0.0082, both statistically significant after B–H correction at FDR 0.05; Table 2). Comparing the current cohort with the initially reported p.Met992del individuals,^4^ the *overall* frequency of abnormal development with/without learning disabilities was significantly higher in the current study (38.8% versus 17%; *P* = 0.0066; Table S7), even after excluding the individuals with genomic imbalances that may have a modifying effect (36.8% versus 17%; *P* = 0.0160; Table S7).

## Discussion

A renewed interest in NF1 genotype–phenotype correlations is emerging, especially with the rapid development and accessibility of genomic technology, given the recent identification of four clinically significant genotype–phenotype correlations.^4–7,27^ The constitutional *NF1* microdeletion and *NF1* missense pathogenic variants affecting codons 844–848 are important risk factors for severe presentation, including a high prevalence of plexiform neurofibromas at an earlier age, dysmorphic facial features and global developmental delay with/without learning disabilities and an increased lifetime risk for malignant peripheral nerve sheath tumors (MPNSTs) in individuals with the *NF1* microdeletion,^27^ and a significant increase in number of plexiform and symptomatic spinal neurofibromas, symptomatic OPGs, skeletal abnormalities, and malignant neoplasms in individuals with the *NF1* missense pathogenic variants at residues 844–848^7^. Furthermore, a mild clinical presentation lacking any externally visible plexiform, cutaneous, or subcutaneous neurofibromas is observed in NF1-affected individuals heterozygous for the *NF1* missense pathogenic variants at codon 1809^5,6^ or the 3-bp in-frame *NF1* deletion, c.2970_2972del (p.Met992del).^4^ Because the latter genotype–phenotype correlation was established on a limited number of NF1-affected adults (19/47), an update of this initial intragenic genotype–phenotype correlation is important for clinical practice.

The frequency of the p.Met992del pathogenic variant in the *NF1* mutation–positive unrelated individuals from the UAB cohort is ~0.9% (74/8400), making it one of the most common recurring variants observed in the UAB database. This variant was reported in publicly available disease databases (last accessed July 2018), such as the Leiden Open Variation Database (LOVD; 28 times, 27/28 and 1/28 classified as pathogenic and likely pathogenic, respectively), ClinVar (7 times, all classified as pathogenic) and the Human Gene Mutation Database (HGMD; classified as pathogenic) and absent in population databases, 1000 Genomes, and the Exome Variant Server (EVS), except for a single report in the Genome Aggregation Database (gnomAD; the variant’s frequency in all populations is 0.00041%), and completely segregated with the disorder in 14 affected individuals from 12 unrelated families and was proven to be de novo in six probands. As such, the *NF1* deletion, c.2970_2972del (p.Met992del), should undoubtedly be classified as pathogenic according to current recommendations.^28^

Cutaneous and subcutaneous neurofibromas are benign tumors located on or just under the skin, typically developing around puberty, and almost all NF1-affected adults have at least several of them.^16^ Over the course of time, the number of neurofibromas usually increases, varying from hundreds to thousands. Plexiform neurofibromas may be recognized earlier as they may occur congenitally, but most of them grow slowly, with externally visible plexiform neurofibromas becoming apparent in the early years of life. Plexiform neurofibromas arise in peripheral nerves and are clinically suspected in 15–30% of the NF1-affected general population, but the prevalence of the internal tumors is higher.^3,11,18,29–31^ Moreover, plexiform and subcutaneous neurofibromas are associated with an increased lifetime risk for the development of MPNSTs, resulting in significant morbidity for these individuals.^32–34^ From a clinical point of view, it is important to identify early those individuals with an increased risk to develop malignancies so as to provide them personalized management and genetic counseling.

In the current study, we confirmed the paucity of superficial plexiform (0/84 ≥9 years) and cutaneous neurofibromas (0/38 ≥19 years), in line with the original report.^4^ None of the individuals had externally visible plexiform neurofibromas or *histopathologically* confirmed cutaneous or subcutaneous neurofibromas (all *P* < 0.0001, statistically significant at FDR of 0.01 after B–H correction when compared with the cohort of individuals with the *NF1* missense pathogenic variants affecting codons 844–848 and the “classic” NF1-affected population, Table 2). Combining data from this and previous studies^4,25,26^ for plexiform (0/127 ≥9 years), cutaneous, and subcutaneous neurofibromas (0/59 and 0/37 ≥19 years, respectively), we estimate in post hoc power calculation that these sample sizes would allow to detect the presence of plexiform, cutaneous, and subcutaneous neurofibromas with a prevalence of at least 3%, 7%, and 10%, respectively with a power of 95%. However, we cannot speculate about the risk for internal neurofibromas in this cohort as MRI screening was not routinely done in most of the asymptomatic individuals.

The presence of *possible* few cutaneous or subcutaneous lesions was initially mentioned on the phenotypic checklist in 14 individuals ≥14 years, but upon further detailed clinical evaluation by NF1 experts and/or histopathological analysis the lesions were diagnosed as lipomas in 5/14 individuals (for the remaining cases no follow-up was available) (see Comments in Table S1 and Table S4). Moreover, in none of the individuals were additional lesions observed over time, further supporting that these lesions were unlikely to be neurofibromas, because cutaneous and subcutaneous neurofibromas usually increase in number over the years. The *overall* prevalence of lipomas in the p.Met922del-positive individuals from this and a previous study^4^ was 5.5% (7/128); lipomas have also been observed in ~20% of individuals (21/115) with Legius syndrome (MIM 611431), another mild phenotype consisting of pigmentary spots only, caused by pathogenic variants in *SPRED1* (MIM 609291).^35^

Besides neurofibromas and OPGs, NF1-affected individuals may develop other benign and malignant tumors, such as MPNSTs, rhabdomyosarcomas, leukemias, neuroblastomas, pheochromocytomas, gastrointestinal stromal tumors (GISTs), glomus tumors, and breast and/or ovarian cancer^36^ that depending on the clinical and histological grade may significantly increase mortality in the NF1 population.^1^ Nonoptic gliomas are one of the most common brain tumors in NF1-affected individuals, with an overall frequency greater than 100 times that in the general population.^37^ In the current study, we reported 6/126 (4.8%) individuals with nonoptic brain tumors, mostly low-grade and asymptomatic (Table S1 and Table S5). The prevalence in the studied cohort was similar to that recorded in the recent report on nonoptic gliomas by Sellmer et al.^38^ (24/562, 4.3%). Additionally, we confirmed the presence of the *NF1* p.Met992del pathogenic variant with LOH in three astrocytomas from a single case (UAB-R5571-F), confirming these as NF1-associated tumors. Burgoyne et al.^26^ also demonstrated the occurrence of a germline p.Met992del along with a somatic *NF1* p.Ser1407fs*21 pathogenic variant in an individual with multiple CALMs and mild axillary freckling who developed a low-grade GIST. Notwithstanding the above, brain tumors seem to be the most common complications in individuals heterozygous for the constitutional *NF1* p.Met992del. Even though brain tumors in NF1 usually are low-grade lesions and have a more benign course than in the individuals without NF1^39^, it is important that clinicians involved in the care of individuals with NF1 are aware of this complication.

The highly variable inter- and intrafamilial expressivity and age-dependency of most symptoms undoubtedly hampers the accurate NF1 clinical diagnosis, especially in infants and individuals with mosaic NF1. Indeed, nearly half of sporadic NF1-affected children do not fulfill the NIH diagnostic criteria by 1 year of age.^3^ In this study, we observed that 14/60 (23.3%) of individuals ≥9 years did not fulfill the NIH diagnostic criteria. However, these numbers increase dramatically if the family history is *not* taken into account (23/60, 38.3%), which necessarily applies to all sporadic cases (Table S8). An important reason why the p.Met992del-positive individuals do not fulfill NIH criteria is the absence of any externally visible neurofibromas, even in adults. As only 10/67 (14.9%) of individuals ≥9 years developed Lisch nodules (Table 1), a systematic ophthalmological examination for Lisch nodules may not be of major help in making an early clinical diagnosis in the p.Met992del-positive individuals. The cumulative advances in our understanding of NF1 prompt the need to consider if additional clinical signs, such as juvenile xanthogranulomas (JXG), nevus anemicus (NA), or choroidal abnormalities, as well as proof of a pathogenic variant, may help to establish an early NF1 diagnosis, especially in children.^40^ However, screening for JXG, NA, and choroidal abnormalities was not routinely done in the current study.

Cognitive impairment and/or learning disabilities are part of the phenotype associated with the *NF1* p.Met992del, as these were present in 50/129 individuals (38.8%), including five children with severe global developmental delay and/or gross motor delay (UAB-R1542, UAB-R1873, UAB-R4613, UAB-R6975, and UAB-R4846). In some cases, aCGH analysis was performed in addition to the *NF1* molecular analysis, identifying genomic imbalances that may have modifying roles (Table S1). One of these cases (UAB-R6975), a 9-year-old girl with serious neurocognitive issues (severely deficient verbal intellectual function, impaired expressive and receptive language delays, and features of autism spectrum disorder, generalized anxiety disorder, and dyspraxia), was found to have a constitutional 15q13.3 duplication, encompassing the *CHRNA7* (MIM 118511) and *OTUD7A* (MIM 612024) genes (Table S1). *CHRNA7* duplication is however of unknown significance, although cognitive impairment and psychiatric disorders have been observed in several families with such microduplications.^41^ On the other hand, another individual (UAB-R4613) also with very severe developmental delay (inability to read and write at age 17 and only processing basic information, such as following directions and completing small assignments) had a normal aCGH result. Nevertheless, compared with the initial study,^4^ abnormal development with/without learning disabilities was much more common in the current study (*P* = 0.0066; Table S7) and requires special attention.

Furthermore, Noonan-like phenotype and pulmonic stenosis were more frequent in the studied cohort compared with the general NF1 population (*P* < 0.0001 and *P* = 0.0009, respectively; both statistically significant at FDR of 0.01 after B–H correction); however, 2/15 individuals showed presence of the 1q21.1 microdeletion syndrome (Fig. S2), which is associated with some Noonan-like features. Pathogenic or likely pathogenic variants in *PTPN11* (MIM 176876) and/or other Noonan-related disorder genes were ruled out in 13/15 cases with Noonan-like phenotype; two individuals (EUR-R5 and EUR-R19) were not tested (Table S1).

Although the initial genotype–phenotype report, demonstrating very mild phenotype in the *NF1* p.Met992del-positive individuals, was published a decade ago,^4^ the biological reasons why these specific individuals do not develop any externally visible neurofibromas are still unclear. Methionine at codon 992 is surrounded by evolutionarily highly conserved amino acids, but no significant progress has been made toward better understanding of this region of the protein. Besides the well-understood role of the GAP-related domain, only a few functional studies have verified how other domains regulate the function of neurofibromin.^42^ Therefore, there is a need to increase efforts to develop functional assays to improve our understanding of the biological effect of this and other pathogenic variants. Understanding the molecular mechanisms whereby p.Met992del is *not* associated with the development of neurofibromas in NF1-affected individuals may help to identify new therapeutic targets.

The mild phenotype described herein with mainly pigmentary manifestations only is not limited to NF1 because several other conditions with overlapping features, especially Legius syndrome and Noonan syndrome with multiple lentigines (formerly called LEOPARD syndrome, MIM 151100), may be phenotypically/clinically indistinguishable from the p.Met992del or p.Arg1809 phenotypes. As such, the 2016 American Association for Cancer Research (AACR) Childhood Cancer Predisposition workshop recommends that children fulfilling one or more NIH diagnostic criteria have the NF1 diagnosis molecularly confirmed.^43^ Establishing a correct NF1 diagnosis, especially in young individuals who do not meet the clinical criteria, is crucial in determining appropriate clinical management. Though genotype–phenotype correlations are exceptions in NF1, the identification of clinically relevant genotype–phenotype correlations facilitates counseling and surveillance of a significant number of NF1 patients.

In conclusion, we clearly confirmed that a 3-bp in-frame deletion of the *NF1* gene, c.2970_2972del (p.Met992del), is associated with a mild phenotype lacking externally visible plexiform, cutaneous, or subcutaneous neurofibromas. Through the analysis on a well-described cohort of 135 individuals (including 90 individuals ≥9 years) we bring to attention the significant risk for cognitive impairment, learning disabilities, and nonoptic brain tumors associated with this particular *NF1* genotype. Therefore, clinicians specializing in the care of NF1-affected individuals should be aware of these complications, mostly occurring in oligosymptomatic individuals who may, nevertheless, require personalized attention for pathogenic variant-specific complications.

## URLs

1000 Genomes: http://www.internationalgenome.org/

ClinVar: https://www.ncbi.nlm.nih.gov/clinvar/

gnomAD Browser: http://gnomad.broadinstitute.org/

GraphPad: https://www.graphpad.com/

HGMD: http://www.hgmd.cf.ac.uk/ac/index.php

LOVD: https://databases.lovd.nl/shared/genes/NF1

Exome Variant Server: http://evs.gs.washington.edu/EVS/

OMIM: http://www.omim.org/

## Electronic supplementary material

Supplementary Information

Supplementary Data
