# Supplementary material for: Expanding the clinical phenotype of individuals with a 3-bp in-frame deletion of the NF1 gene (c.2970_2972del): an update of genotype–phenotype correlation
Source: Genet Med. 2018 Sep 7;21(4):867–76. doi: 10.1038/s41436-018-0269-0 (PMC6752285; doi:10.1038/s41436-018-0269-0)
Supplement: Supplementary file 1 — Supplementary Information [file 41436_2018_269_MOESM1_ESM.pdf]

Supplementary Information for the article

Expanding the clinical phenotype of individuals with a 3-bp in-frame deletion of the *NF1* gene (c.2970_2972del): an update of genotype-phenotype correlation.

Magdalena Koczkowska, PhD, Tom Callens, BSc, Alicia Gomes, MS, CGC, Angela Sharp, MMedSc, Yunjia Chen, PhD, Alesha D. Hicks, BSc, Arthur S. Aylsworth, MD, Amedeo A. Azizi, MD, Donald G. Basel, MD, Gary Bellus, MD, PhD, Lynne M. Bird, MD, Maria A. Blazo, MD, Leah W. Burke, MD, Ashley Cannon, PhD, CGC, Felicity Collins, PhD, Colette DeFilippo, MS, LCGC, Ellen Denayer, MD, PhD, Maria C. Digilio, MD, Shelley K. Dills, MS, CGC, Laura Dosa, MD, Robert S. Greenwood, MD, Cristin Griffis, MS, CGC, Punita Gupta, MD, Rachel K. Hachen, MD, MPH, Concepción Hernández-Chico, PhD, Sandra Janssens, MD, PhD, Kristi J. Jones, MBBS, Justin T. Jordan, MD, MPH, Peter Kannu, MB ChB, PhD, Bruce R. Korf, MD, PhD, Andrea M. Lewis, CGC, Robert H. Listernick, MD, Fortunato Lonardo, MD, Maurice J. Mahoney, MD, JD, Mayra Martinez Ojeda, MD, Marie T. McDonald, MD, Carey McDougall, MS, CGC, Nancy Mendelsohn, MD, David T. Miller, MD, PhD, Mari Mori, MD, Rianne Oostenbrink, MD, PhD, Sebastién Perreault, MD, Mary Ella Pierpont, MD, PhD, Carmelo Piscopo, MD, PhD, Dinel A. Pond, MS, CGC, Linda M. Randolph, MD, Katherine A. Rauen, MD, PhD, Surya Rednam, MD, S. Lane Rutledge, MD, Veronica Saletti, MD, G. Bradley Schaefer, MD, Elizabeth K. Schorry, MD, Daryl A. Scott, MD, PhD, Andrea Shugar, MS, CGC, Elizabeth Siqveland, APRN, CNP, Lois J. Starr, MD, Ashraf Syed, MD, Pamela L. Trapane, MD, Nicole J. Ullrich, MD, PhD, Emily G. Wakefield, CGC, Laurence E. Walsh, MD, Michael F. Wangler, MD, Elaine Zackai, MD, Kathleen B.M. Claes, PhD, Katharina Wimmer, PhD, Rick van Minkelen, PhD, Alessandro De Luca, PhD, Yolanda Martin, PhD, Eric Legius, MD, PhD, Ludwine M. Messiaen, PhD

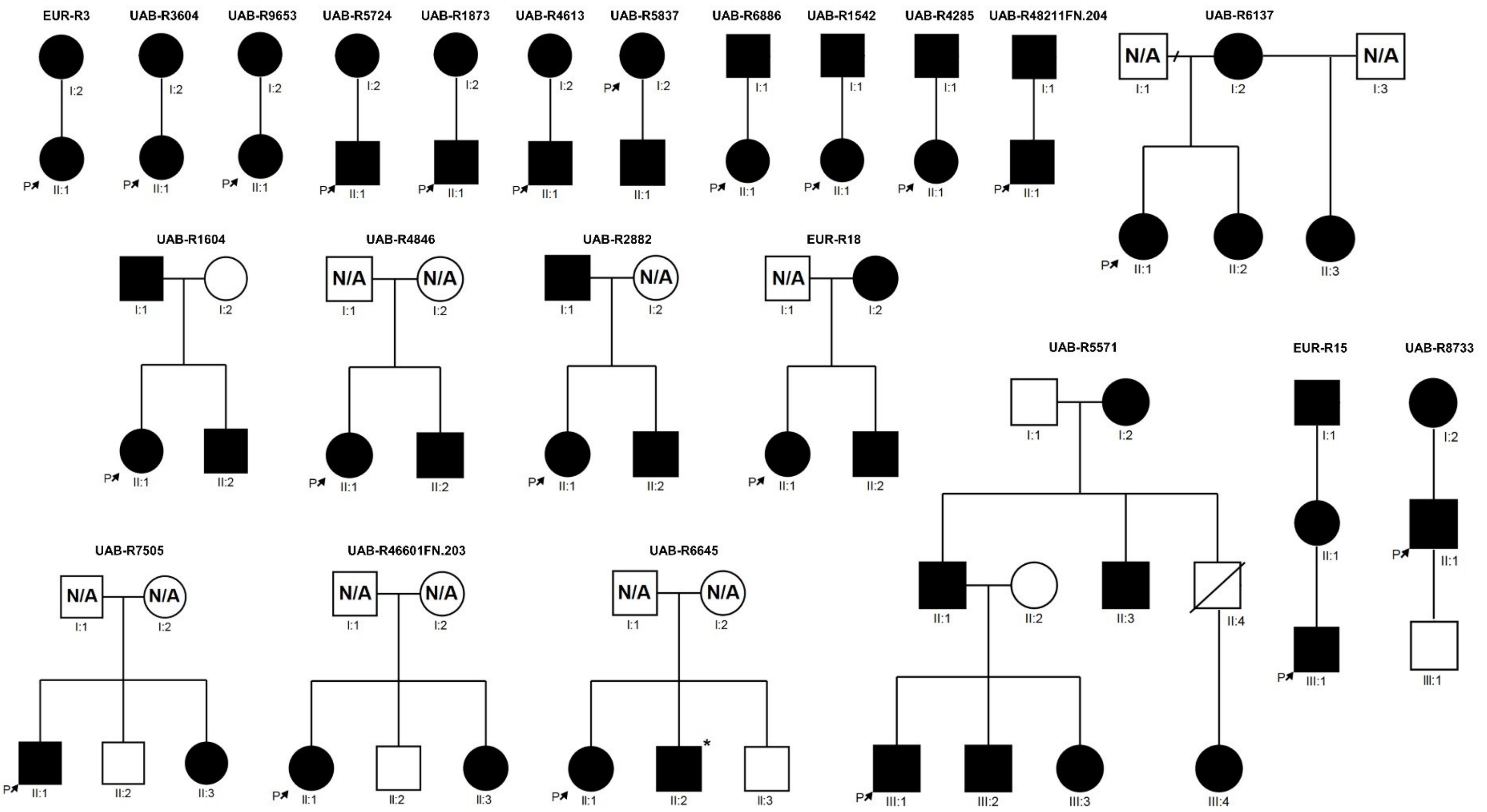

Figure S1. Pedigrees of the familial cases in the current study.

The filled black symbols represent the individuals who are positive for a single *NF1* amino acid deletion (p.Met992del), while the open symbols represent relatives who are negative for the family specific variant. The arrow indicates the proband of the family. For the first generation, father is always I:1 and mother is I:2.

Abbreviations: **N/A** - the relatives not tested for *NF1* specific variant; ***** - no clinical information available for these individuals.

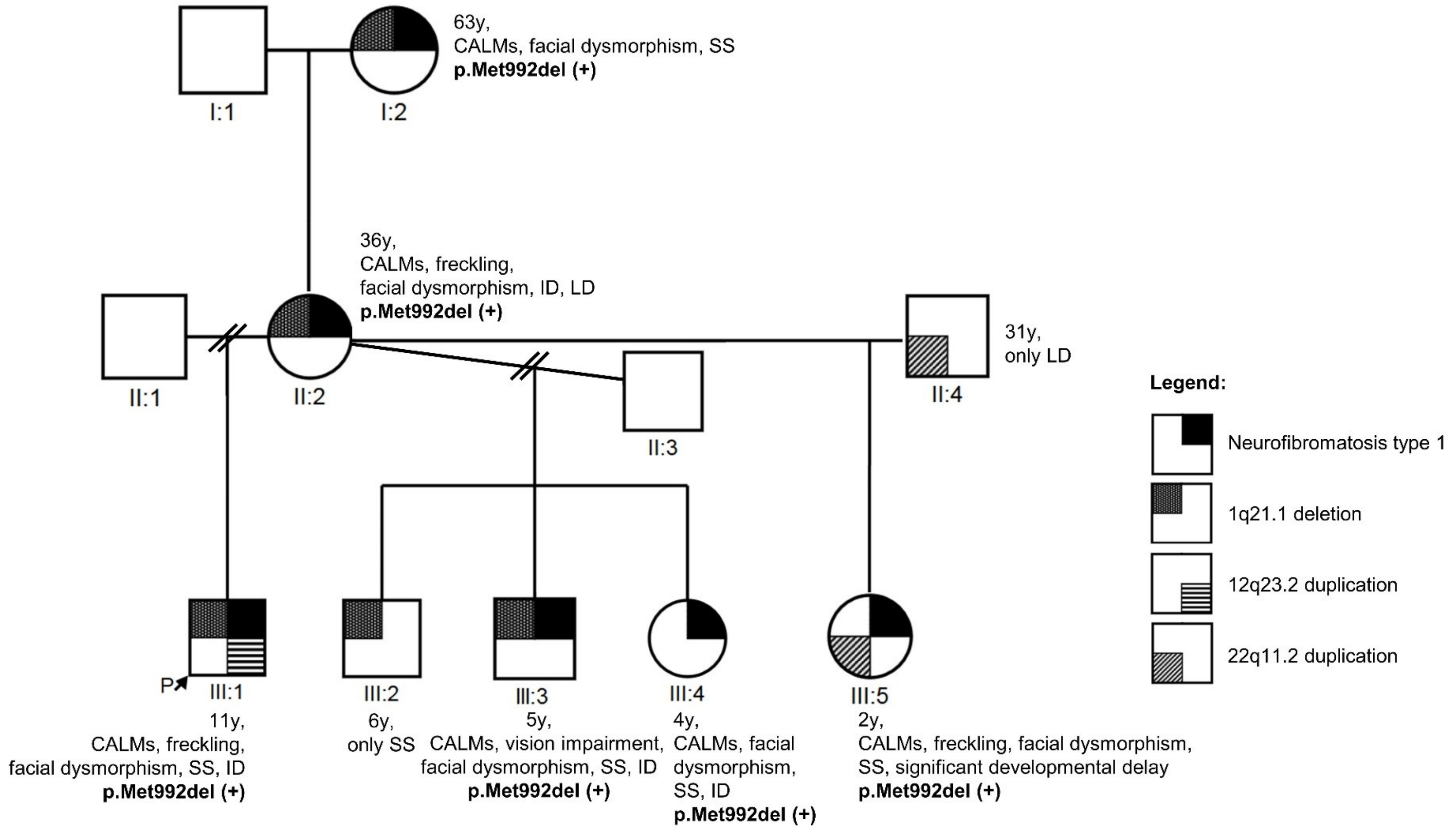

Figure S2. Detailed complex pedigree of family (UAB-R1873) with three different conditions (neurofibromatosis type 1, 1q21.1 deletion and 22q11.2 duplication syndromes) and an additional variant of unknown significance (12q23.2 duplication) segregating among relatives.

Abbreviations: SS - short stature; **ID** - intellectual disability; **LD** - learning disability.

Table S1. Clinical details for 135 individuals with the *NF1* p.Met992del pathogenic variant from 103 different families.

Table S1 is included as a separate Excel file.

[A] For individuals with one asterisk (*) the standardized phenotypic checklist forms were not re-verified/updated by the referring physicians, the data are based on the originally submitted forms; individuals with two asterisks (**) had incomplete phenotypic checklist forms.

[B] **UAB**: **U**niversity of **A**labama at **B**irmingham (74 probands and 27 relatives); **EUR**: individuals referred by the **Eur**opean collaborators (29 probands and 5 relatives), including Medical University of Vienna, Vienna, Austria and Medical University of Innsbruck, Austria (2 probands), Hospital Universitario Ramón y Cajal, Institute of Health Research (IRYCIS) and Center for Biomedical Research-Network for Rare Diseases (CIBERER), Madrid, Spain (2 probands and 1 relative), IRCCS Foundation, Carlo Besta Neurological Institute, Milan, Italy (1 proband), Erasmus Medical Center, Rotterdam, the Netherlands (9 probands), Molecular Genetics Unit, Casa Sollievo della Sofferenza Hospital, IRCCS, San Giovanni Rotondo, Italy (4 probands and 4 relatives), University Hospital, Ghent, Belgium (6 probands) and University of Leuven, Leuven, Belgium (5 probands).

[C] **F**: **f**amilial; **PrS**: **pr**oven **s**poradic; **RS**: **r**eportedly **s**poradic; **NS**: **n**ot **s**pecified; **UN**: **un**known

[D] Exact age was used to calculate Height and Head Circumferences (HC), but provided as age-groups in Table S1: 0-24 months; 2-4 years; 5-8 years; 9-13 years; 14-18 years; 19-26 years; >26 years.

[E] **F**: **f**emale; **M**: **m**ale

[F] **W**: **W**hite; **His**: **His**panic; **AA**: **A**frican **A**merican; **As**: **As**ian; **NaA**: **N**ative **A**merican; **A**: **A**frican; **O:** **O**ther

[G] An individual was classified as having Noonan-like phenotype if at least two of the following features were present: short stature (SS), low set ears (LSE), hypertelorism (HTL), ptosis (PT), midface hypoplasia (MH), webbed neck (WN) or pulmonic stenosis (PS). List of 16 Noonan-related disorder genes tested in all 10 individuals with Noonan-like phenotype referred to UAB: *PTPN11* (MIM: 176876), *SPRED1* (MIM: 609291), *BRAF* (MIM: 164757), *CBL* (MIM: 165360), *HRAS* (MIM: 190020), *KRAS* (MIM: 190070), *MAP2K1* (MIM: 176872), *MAP2K2* (MIM: 601263), *NRAS* (MIM: 164790), *RAF1* (MIM: 164760), *SHOC2* (MIM: 602775), *SOS1* (MIM: 182530), *RIT1* (MIM: 609591), *RASA2* (MIM: 601589), *SOS2* (MIM: 601247) and *PPP1CB* (MIM: 600590).

[H] Height percentiles for Hispanic and Asian individuals were provided in square brackets to indicate that they were excluded from the data analysis on frequency of short or normal stature due to the lack of ethnic-specific growth charts.

[I] Individuals with ADD/ADHD, but normal development were still classified as normal.

Abbreviations:

NS: **n**ot **s**pecified, i.e. no value provided on the phenotypic checklist; **UN**: **un**kown; **CALMs**: **c**afé-**a**u-**l**ait **m**acule**s**; **bil**: **bil**ateral; **gr**: **gr**oin; **ax**: **ax**illary; **sub**: **sub**mammary; **OPG**: **o**ptic **p**athway **g**lioma; **N, UN**: **n**o symptomatic OPG or symptomatic spinal neurofibroma, **un**known if any asymptomatic OPG or asymptomatic spinal neurofibromas are present; **N, MRI**: **n**o symptomatic and asymptomatic OPG or spinal neurofibroma detected by **MRI**; **Y, MRI**: **y**es, presence of OPG or spinal neurofibroma confirmed by **MRI**; **HTL**: **h**yper**t**e**l**orism; **WN**: short/**w**ebbed **n**eck; **MH**: **m**idface **h**ypoplasia; **LSE**: **l**ow **s**et **e**ars; **LPH**: **l**ow **p**osterior **h**airline; **SS**: **s**hort **s**tature; **PT**: **pt**osis; **PS**: **p**ulmonic **s**tenosis; **DPF**: **d**ownslanting **p**alpebral **f**issures; **LD**: **l**earning **d**isability; **ADD**: **a**ttention **d**eficit **d**isorder; **ADHD**: **a**ttention **d**eficit **h**yperactivity **d**isorder; **SD**: **s**peech **d**elay; **FSIQ**: **F**ull **S**cale **I**ntelligence **Q**uotient; **F**: **f**ather: **M**: **m**other; **S**: **s**ibling; **C**: **c**hild; **MGF**: **m**aternal **g**rand**f**ather, **PGM**: **p**aternal **g**rand**m**other; **PU**: **p**aternal **u**ncle; **PC**: **p**aternal **c**ousin; **HS**: **h**alf **s**ibling; **HC**: **h**ead **c**ircumference; **aCGH**: **a**rray **c**omparative **g**enomic **h**ybridization; **MRI**: **m**agnetic **r**esonance **i**maging; **VUS**: **v**ariant of **u**ncertain **s**ignificance; **DNET**: **d**ysembryoplastic **n**euro**e**pithelial **t**umor; **GIST**: **g**astro**i**ntestinal **s**tromal **t**umor; **GERD**: **g**astro**e**sophageal **r**eflux **d**isease; **WHO**: **W**orld **H**ealth **O**rganization.

Table S2. List of all adjusted *P* values after applying the Benjamini-Hochberg (B-H) correction for multiple testing with false discovery rates (FDR) at 0.05 and 0.01.

***P* value**	**index**	**Statistical significance at FDR=0.05**		**Statistical significance at FDR=0.01**	
		**B-H critical [a]**	**value [b]**	**B-H critical [a]**	**value [b]**
1.0000	1	0.0250		0.0050	
1.0000	2	0.0245		0.0049	
1.0000	3	0.0240		0.0048	
1.0000	4	0.0235		0.0047	
1.0000	5	0.0230		0.0046	
1.0000	6	0.0225		0.0045	
1.0000	7	0.0220		0.0044	
0.8778	8	0.0215		0.0043	
0.8414	9	0.0210		0.0042	
0.7365	10	0.0205		0.0041	
0.5838	11	0.0200		0.0040	
0.5628	12	0.0195		0.0039	
0.5613	13	0.0190		0.0038	
0.5336	14	0.0185		0.0037	
0.5255	15	0.0180		0.0036	
0.5193	16	0.0175		0.0035	
0.4922	17	0.0170		0.0034	
0.3261	18	0.0165		0.0033	
0.2982	19	0.0160		0.0032	
0.2035	20	0.0155		0.0031	
0.1934	21	0.0150		0.0030	
0.1388	22	0.0145		0.0029	
0.0785	23	0.0140		0.0028	
0.0640	24	0.0135		0.0027	
0.0553	25	0.0130		0.0026	
0.0473	26	0.0125		0.0025	
0.0357	27	0.0120		0.0024	
0.0267	28	0.0115		0.0023	
0.0159	29	0.0110		0.0022	
0.0111	30	0.0105		0.0021	
**0.0082**	31	0.0100	*	0.0020	
0.0056	32	0.0095	*	0.0019	
0.0033	33	0.0090	*	0.0018	
0.0018	34	0.0085	*	0.0017	
0.0016	35	0.0080	*	0.0016	
**0.0009**	36	0.0075	*	0.0015	*
0.0005	37	0.0070	*	0.0014	*
0.0001	38	0.0065	*	0.0013	*
0.0001	39	0.0060	*	0.0012	*
0.0001	40	0.0055	*	0.0011	*
0.0001	41	0.0050	*	0.0010	*
0.0001	42	0.0045	*	0.0009	*
0.0001	43	0.0040	*	0.0008	*
0.0001	44	0.0035	*	0.0007	*
0.0001	45	0.0030	*	0.0006	*
0.0001	46	0.0025	*	0.0005	*
0.0001	47	0.0020	*	0.0004	*
0.0001	48	0.0015	*	0.0003	*
0.0001	49	0.0010	*	0.0002	*
0.0001	50	0.0005	*	0.0001	*

[a] According to Thissen et al. (2002)[4]; [b] An asterisk (*) indicates the statistically significant *P*-value after B-H correction.

Table S3. Clinical characterization of 31 individuals who did not fulfill the current NIH diagnostic criteria, based on physical features, not taking family history into account.

ID [a]	Family history	Age, years	CALMs	Freckles	Lisch nodules	Neurofibromas	OPG	Specific skeletal abnormalities [b]	NIH diagnostic criteria [c]
UAB-R1031	RS	<2	>5	-	-	-	N, MRI	-	1/6
UAB-R4846-S	F	<2	>5	-	-	-	N, UN	-	1/6
UAB-R8467	RS	<2	>5	-	-	-	N, UN	-	1/6
UAB-R7536	F	<2	>5	-	-	-	N, UN	-	1/6
UAB-R3604	F	2-4	>5	-	-	1 possible subcutaneous nf [d]	N, UN	-	1/6
UAB-R5724	F	2-4	>5	-	-	2 possible subcutaneous nf [d]	N, UN	-	1/6
UAB-R4846	F	2-4	>5	-	-	-	N, UN	-	1/6
UAB-R8655	NS	5-8	>5	-	-	-	N, UN	-	1/6
UAB-R9147	F	5-8	>5	-	-	-	N, MRI	-	1/6
UAB-R3067	F	5-8	>5	-	-	-	N, UN	-	1/6
EUR-R21	UN	5-8	>5	-	-	-	N, MRI	-	1/6
UAB-R6764	RS	9-13	>5	-	-	-	N, UN	-	1/6
EUR-R2	RS	9-13	>5	-	-	-	N, MRI	-	1/6
UAB-R5571-S1	F	14-18	>5	-	-	-	N, MRI	-	1/6
UAB-R5571-S2	F	14-18	>5	-	-	-	N, UN	-	1/6
UAB-R2542	F	14-18	>5	-	-	-	N, UN	-	1/6
UAB-R8733	F	14-18	>5	-	-	-	N, UN	-	1/6
UAB-R9815	UN	14-18	>5	-	-	1 subcutaneous nf [d]	N, UN	-	1/6
UAB-R5725	F	14-18	>5	-	-	-	N, UN	-	1/6
UAB-R46601FN.203	F	14-18	>5	-	-	-	N, UN	-	1/6
EUR-R6	UN	14-18	<6	+	-	-	N, MRI	-	1/6
UAB-R5571-PC	UN	19-26	>5	-	-	-	N, MRI	-	1/6
**UAB-R6643**	RS	19-26	<6	-	-	-	N, MRI	-	0/6
**UAB-R9653-M**	NS	19-26	<6	-	-	1 possible subcutaneous nf [d]	N, UN	-	0/6
**UAB-R3604-M**	F	19-26	<6	-	-	1 cutaneous nf [d]	N, UN	-	0/6
EUR-R4	RS	19-26	>5	-	-	-	N, MRI	-	1/6
**EUR-R7**	F	19-26	<6	-	-	1 cutaneous nf [d]	N, MRI	-	0/6
EUR-R15	F	19-26	>5	-	-	-	N, MRI	-	1/6
UAB-R4285-F	F	>26	<6	-	+	-	N, UN	-	1/6
**UAB-R19915211**	RS	>26	<6	-	-	-	N, MRI	-	0/6
UAB-R48211FN.204-F	UN	>26	>5	-	-	-	N, UN	-	1/6

[a] IDs of individuals with none of the diagnostic criteria of NF1 fulfilled were bolded; [b] Only skeletal abnormalities with sphenoid dysplasia or thinning of the long bone cortex with/without pseudarthrosis have been taken into account; [c] Only clinical features without the presence/absence of a first-degree relative that meets NIH criteria have been taken into account; [d] None were biopsied and therefore none have been histologically confirmed.

Abbreviations: F - **f**amilial; **RS** - **r**eportedly **s**poradic; **NS** - **n**ot **s**pecified; **UN** - **un**known; **CALMs** - **c**afé-**a**u-**l**ait **m**acule**s**; **OPG** - **o**ptic **p**athway **g**lioma; **N, UN** - **n**o symptomatic OPG, **un**known if any asymptomatic OPG is present; **N, MRI** - **n**o symptomatic and asymptomatic OPG detected by **MRI**; **nf** - **n**euro**f**ibroma(s).

Table S4. Cutaneous and/or subcutaneous lesions in individuals heterozygous for the *NF1* p.Met992del pathogenic variant.

ID	Age, years	Cutaneous and/or subcutaneous lesions	Comment(s)
UAB-R3604	2-4	1 *possible* subcutaneous neurofibroma, no histopathological confirmation	-
UAB-R5724	2-4	2 small *possible* subcutaneous neurofibromas, no histopathological confirmation	-
UAB-R9815	14-18	1 subcutaneous neurofibroma, no histopathological confirmation	-
UAB-R6137-HS	14-18	1 subcutaneous neurofibroma, no histopathological confirmation	-
UAB-R2882-S	14-18	6 lesions, located on back, flank and hip areas, initially diagnosed as *possible* neurofibromas (all lesions were surgically removed)	the diagnosis of **lipomas** (for 5/6 lesions) and **angiolipoma** (for 1/6 lesion) was established after the detailed histopathological evaluation
UAB-R9653-M	19-26	1 *possible* subcutaneous neurofibroma, no histopathological confirmation	-
UAB-R3604-M	19-26	1 cutaneous neurofibroma, no histopathological confirmation	-
EUR-R4	19-26	1 lesion at right shoulder, initially diagnosed as neurofibroma	the diagnosis of **lipoma** was established after the detailed histopathological evaluation
EUR-R7	19-26	1 cutaneous neurofibroma, no histopathological confirmation	-
UAB-R8733-M	>26	2-6 subcutaneous neurofibromas, no histopathological confirmation	-
UAB-R4285	>26	2-6 subcutaneous neurofibromas, no histopathological confirmation	-
UAB-R6137-M	>26	2-6 subcutaneous neurofibromas, no histopathological confirmation	-
EUR-R15-M	>26	1 lesion on leg, initially diagnosed as neurofibroma	the diagnosis of **lipoma** was established after the detailed clinical evaluation by a geneticist and dermatologist
EUR-R15-MGF	>26	skin lesions (the exact number was not available)	the diagnosis of **lipomas** was established after the detailed histopathological evaluation
EUR-R19	>26	multiple subcutaneous lesions	the diagnosis of **lipomas** was established after the detailed clinical evaluation by an NF1 expert
EUR-R26	>26	2-6 cutaneous neurofibromas, no histopathological confirmation	-

Table S5. Neoplasms in individuals heterozygous for the *NF1* p.Met992del pathogenic variant.

ID	Family history	Age, years	CALMs	Freckles	Others	Comment(s)
**LIPOMA(S)**						
UAB-R2882-S	F	14-18	<6	NS	none	**LIPOMAS** and **ANGIOLIPOMA** (*histopathologically confirmed*)
EUR-R4	RS	19-26	>5	-	-	**LIPOMA** (*histopathologically confirmed*)
EUR-R15-M	F	>26	>5	+	-	**LIPOMA** on leg
EUR-R15-MGF	F	>26	>5	NS	cardiovascular disease present, but no details available	**LIPOMAS** (*histopathologically confirmed*)
EUR-R19	RS	>26	>5	UN	Noonan-like phenotype, LD, gastro-esophageal reflux, severe postural tremor of both hands, bilateral carpal tunnel syndrome and psychiatric problems	multiple subcutaneous **LIPOMAS**
**BRAIN TUMORS**						
UAB-R2604	PrS	9-13	>5	+	-	**HYPOTHALAMIC GLIOMA**
EUR-R24	NS	9-13	>5	+	short stature	low-grade **ASTROCYTOMA** or **DNET** (dysembryoplastic neuroepithelial tumors) - differential diagnosis required (see Comments in Table S1)
UAB-R1262	RS	14-18	>5	+	Noonan-like phenotype, macrocephaly, LD, motor apraxia	**OLIGODENDROGLIOMA** (asymptomatic, surgically removed, *histopathologically confirmed*)
EUR-R16	RS	14-18	>5	+	Lisch nodules, scoliosis, bilateral club feet, sinus arrhythmia and atypia of repolarization, abnormal development, LD	**BRAIN TUMOR with hamartomatous aspect by MRI of the encephalon**
EUR-R15	F	19-26	>5	-	short stature, macrocephaly, panhypopituitarism secondary to the operation	**CRANIOPHARYNGIOMA** (asymptomatic, surgically removed, *histopathologically confirmed*)
UAB-R5571-F	F	>26	-	-	no clinical signs of NF1	three **JUVENILE PILOCYTIC ASTROCYTOMAS** surgically removed (brainstem and top left site of the brain, *histopathologically confirmed and confirmed as NF1-related tumors)*
**OTHERS**						
UAB-R3587	RS	19-26	>5	+	severe scoliosis, short stature, hypertelorism, mild developmental delay, LD	**PILOMYXOID ASTROCYTOMA** of the thoracic spine (low-grade, *histopathologically confirmed*); case reported by Dunn-Pirio et al. (2016)[2]
EUR-R18-M	F	>26	>5	+	scoliosis, pectus abnormality, Noonan-like phenotype, short stature	**NEUROBLASTOMA**; case reported by De Luca et al. (2005)[3]

Abbreviations: F - **f**amilial; **PrS** - **pr**oven **s**poradic; **RS** - **r**eportedly **s**poradic; **NS** - **n**ot **s**pecified; **CALMs** - **c**afé-**a**u-**l**ait **m**acule**s**; **LD** - **l**earning **d**isability.

Table S6. Plexiform, cutaneous and subcutaneous neurofibromas in individuals heterozygous for the *NF1* p.Met992del pathogenic variant in this and previous studies: a meta-analysis.

Age group	Number of individuals				
	This study [a]	Upadhyaya et al. (2007)[5]	Quintáns et al. (2011)[6]	Burgoyne et al. (2017)[7]	Total
**PLEXIFORM NEUROFIBROMAS**					
9-13 years	0/27	0/10	-	-	0/37
14-18 years	0/19	0/12	-	-	0/31
19-26 years	0/12	0/3	-	-	0/15
≥26 years	0/26	0/16	0/1	0/1	0/44
**CUTANEOUS NEUROFIBROMAS**					
19-26 years	2/12 (both with 1 neurofibroma)	0/3	-	-	2/15 [b]
27-35 years	1/7 (2-6 neurofibromas)	0/3	-	0/1	1/11 [b]
36-50 years	0/17	0/7	0/1	-	0/25 [b]
>50 years	0/2	0/6	-	-	0/8
**SUBCUTANEOUS NEUROFIBROMAS**					
19-26 years	1/12 (1 *possible* neurofibroma)	ND	-	-	1/12 [b]
27-35 years	0/6	ND	-	0/1	0/7
36-50 years	3/17 (all with 2-6 neurofibromas)	ND	ND	-	3/17 [b]
>50 years	0/1	ND	-	-	0/1

[a] This study included three affected individuals from two-generation family reported previously in De Luca et al. (2005).[3]

[b] Clear-cut multiple cutaneous or subcutaneous neurofibromas were not seen in any individual. Few cases had 1-6 *possible* neurofibromas, but none were biopsied, thus the histopathological diagnosis of neurofibroma was not established. Importantly, for four adults the diagnosis was “lipoma” after detailed clinical and/or histopathological analysis (see Table S4).

Table S7. Comparison of the prevalence of cognitive impairment and/or learning disabilities in the current study and the cohort of individuals with the *NF1* p.Met992del pathogenic variant described previously by Upadhyaya et al. (2007).[5]

NF1 clinical features	Number of individuals (%)		*P* value (2-tailed Fisher's exact test)
	This study	Upadhyaya et al. (2007)[5]	
Cognitive impairment and/or learning disabilities	50/129 (38.8)	8/47 (17)	**0.0066 ↗**
Cognitive impairment and/or learning disabilities after excluding individuals with the additional genomic imbalances [a]	46/125 (36.8)	8/47 (17)	**0.0160 ↗**

[a] After excluding individuals with the additional genomic imbalances besides the *NF1* p.Met992del pathogenic variant, that is UAB-R1873 and UAB-R1873-M with 1q21.1 deletion (MIM: 612474), UAB-R6975 with 15q13.3 duplication and UAB-R8603 with a 16p13 deletion and 14q32.13 duplication (see details in Table S1).

Table S8. Prevalence of individuals with the *NF1* p.Met992del and p.Arg1809 pathogenic variants fulfilling the NIH diagnostic criteria if pigmentary manifestations (i.e. CALMs and/or skinfold freckles) are counted as two independent criteria or a single criterion.

NIH criteria	Number of p.Met992del-positive individuals (%) [a,b]				Number of p.Arg1809-positive individuals (%) [b,c]			
	**≤8 years**	**9-18 years**	**≥19 years**	**Total**	**≤8 years**	**9-18 years**	**≥19 years**	**Total**
Fulfilling the NIH criteria if the family history is taken into account with pigmentary manifestations counted as **two independent criteria**	24/29 (82.8)	34/39 (87.2)	12/21 (57.1)	70/89 (78.7)	18/32 (56.3)	21/23 (91.3)	9/9 (100)	48/64 (75)
Fulfilling the NIH criteria if **solely taking the physical signs into account** with pigmentary manifestations counted as **two independent criteria** [d]	15/29 (51.7)	28/39 (71.8)	9/21 (42.9)	52/89 (58.4)	16/32 (50)	18/23 (78.3)	8/9 (88.9)	42/64 (65.6)
Fulfilling the NIH criteria if the family history is taken into account with pigmentary manifestations counted as **a single criterion**	16/29 (55.2)	23/39 (59)	8/21 (38.1)	47/89 (52.8)	9/32 (28.1)	13/23 (56.5)	5/9 (55.6)	27/64 (42.2)
Fulfilling the NIH criteria if **solely taking the physical signs into account** with pigmentary manifestations counted as **a single criterion** [d]	3/29 (10.4)	7/39 (18)	3/21 (14.3)	13/89 (14.6) [e]	2/32 (6.3)	3/23 (13)	0/9 (0)	5/64 (7.8) [f]

[a] Based on data from the current study.

[b] Only individuals with the complete clinical information, including the ophthalmological results for the presence/absence of Lisch nodules and symptomatic OPGs, have been taken into account.

[c] Based on data from Rojnueangnit et al. (2015).[8]

[d] Only clinical features without the presence/absence of a first-degree relative that meets NIH criteria have been taken into account.

[e] Among 13 individuals with the NF1 clinical diagnosis after combining pigmentary manifestations (i.e. CALMs and/or skinfold freckling) as a single criterion and excluding family history, the features that allowed to fulfill the NIH diagnostic criteria in these individuals included the presence of ≥2 Lisch nodules (10/13), OPG (1/13) and ≥2 subcutaneous neurofibromas (2/13), however, not histopathologically confirmed, thus further clinical and/or histopathological evaluation should have been taken as they likely were misdiagnosed with lipomas.

[f] Among 5 individuals with the NF1 clinical diagnosis after combining pigmentary manifestations (i.e. CALMs and/or skinfold freckling) as a single criterion and excluding family history, the features that allowed to fulfill the NIH diagnostic criteria in these individuals included the presence of ≥2 Lisch nodules (4/5) and tibial dysplasia (1/5).

REFERENCES

1. Hirata Y, Brems H, Suzuki M, et al. Interaction between a domain of the negative regulator of the Ras-ERK pathway, SPRED1 protein, and the GTPase-activating protein-related domain of neurofibromin is implicated in Legius syndrome and neurofibromatosis type 1. *J Biol Chem.* 2016;291:3124-3134.
2. Dunn-Pirio AM, Howell E, McLendon RE, Peters KB. Single-agent carboplatin for a rare case of pilomyxoid astrocytoma of the spinal cord in an adult with neurofibromatosis type 1. *Case Rep Oncol.* 2016;9:568-573.
3. De Luca A, Bottillo I, Sarkozy A, et al. *NF1* gene mutations represent the major molecular event underlying neurofibromatosis-Noonan syndrome. *Am J Hum Genet.* 2005;77:1092-1101.
4. Thissen D, Steinberg L, Kuang D. Quick and easy implementation of the Benjamini-Hochberg procedure for controlling the false positive rate in multiple comparisons. *J Educ Behav Stat.* 2002;27:77-83.
5. Upadhyaya M, Huson SM, Davies M, et al. An absence of cutaneous neurofibromas associated with a 3-bp inframe deletion in exon 17 of the *NF1* gene (c.2970-2972 delAAT): evidence of a clinically significant NF1 genotype-phenotype correlation. *Am J Hum Genet.* 2007;80:140-151.
6. Quintáns B, Pardo J, Campos B, et al. Neurofibromatosis without neurofibromas: confirmation of a genotype-phenotype correlation and implications for genetic testing. *Case Rep Neurol.* 2011;3:86-90.
7. Burgoyne AM, De Siena M, Alkhuziem M, et al. Duodenal-jejunal flexure GI stromal tumor frequently heralds somatic *NF1* and notch pathway mutations. *JCO Precis Oncol.* 2017;1:1-12.
8. Rojnueangnit K, Xie J, Gomes A, et al. High incidence of Noonan syndrome features including short stature and pulmonic stenosis in patients carrying *NF1* missense mutations affecting p.Arg1809: genotype-phenotype correlation. *Hum Mutat.* 2015;36:1052-1063.
